# The source of punishment matters: Third-party punishment restrains observers from selfish behaviors better than does second-party punishment by shaping norm perceptions

**DOI:** 10.1371/journal.pone.0229510

**Published:** 2020-03-02

**Authors:** Hezhi Chen, Zhijia Zeng, Jianhong Ma

**Affiliations:** 1 Department of Psychology and Behavioral Sciences, Zhejiang University, Hangzhou, China; 2 Student Affairs Departments, Zhejiang University of Finance and Economics, Hangzhou, China; Middlesex University, UNITED KINGDOM

## Abstract

Punishment aims to deter individuals’ selfish behaviors, but it can occasionally backfire. Some scholars have proposed promoting prosocial behaviors using punishment that communicates positive social norms because it provides additional motivation. However, it is unclear which factors affect the norm expressive function of punishment. This study proposes that third-party punishment communicates more positive normative information, and thus, promotes more prosocial behavior in observers than does second-party punishment. Using dictator games, we investigated the effects of second-party punishment compared to third-party punishment of another’s unfair sharing on observers’ norm perceptions and subsequent sharing decision-making. Two experiments consistently found that third-party punishment was more effective than second-party punishment at inducing observers’ beliefs that unfair distribution was unusual (descriptive norm) and unacceptable (injunctive norm). The altered descriptive but not injunctive norm perception further guided individuals’ own sharing behaviors. Taken together, these results suggest that third-party punishment might be better than second-party punishment at decreasing selfish behaviors by shaping individuals’ norm perceptions, especially descriptive norm perception, regarding the relevant behaviors.

## Introduction

Punishment is a crucial deterrence strategy against selfish behaviors [1]. Many behavioral experiments have demonstrated that there are fewer selfish behaviors in the presence of punishment opportunities [2–5]. However, punishments might have detrimental effects. For example, the presence of punishment might crowd out an individual’s intrinsic concern for justice [6]. If the costs of punishment are low and/or the likelihood of punishment is low or nonexistent, individuals’ selfish behaviors might increase [7]. Moreover, the detrimental effects of punishment might be avoided when the punishment signals positive social norms [8]. Individuals’ norm perceptions, that is, their beliefs about the manner in which most other people would behave in a given situation (descriptive norm) and the behaviors that most other people would approve in a given situation (injunctive norm), have been found to powerfully influence behavior [9]. Taken together, punishment that expresses positive social norms should increase individuals’ inhibition of selfish behaviors.

Although scholars recognize the importance of the norm expressive function of punishment, the types of punishment that convey positive normative information have not been identified. For example, one factor that might affect the extent to which punishment signals positive social norms is the identity of the punisher. Specifically, punishment inflicted by a third party might have a more positive influence compared to that inflicted by the offended second party on observers’ (uninvolved individuals) perceptions of social norms.

Third-party punishment (TPP), where people punish another’s selfish behavior even when their own interests have not been harmed, is common [10]. However, why would third parties inflict punishment even when it is costly? Some researchers have proposed that people might aim at promoting cooperative behavior that will benefit them in the long run [11]. However, this can hardly explain the high frequency of TPP in the anonymous laboratory setting, where the punisher and punished are unlikely to interact with each other again. Instead, it has been shown that TPP is related to outrage toward the offender [12]. Furthermore, third parties who punish another’s selfish behavior behave more cooperatively than do non-punishers [13]. Consequently, TPP reflects the third party’s moral disapproval of the selfish act and that the third party is unlikely to commit the same transgression.

In contrast, there are many reasons why second parties inflict punishment. Because second parties have been harmed directly, they might inflict punishment to exact revenge. Second-party punishment (SPP) might be inflicted to increase future personal benefits by deterring the offender’s repeated selfish behaviors [14]. Previous empirical studies have found that when an individual’s personal interests are harmed by defection, even those who have defected themselves tend to punish the defector in the same manner as those who have not defected [15, 16].

The signaling theory of punishment suggests that observers are sensitive to such distinctions between TPP and SPP [14, 17]. People generally trust that third-party punishers are willing to cooperate because TPP signals punishers’ concern for the welfare of the victim [13]. Observers are also willing to reward third-party punishers [17]. However, second parties are unlikely to enjoy such benefits from having the reputation of a punisher [14].

In sum, observers of TPP are likely to infer that punishment is driven by a desire for justice, and consequently, will perceive that the selfish behavior is both disapproved by others and generally uncommon. Observers of SPP, however, might infer that the punishment is driven by self-interested motives; thus, their norm perceptions should be less affected.

We conducted two experiments to test whether TPP and SPP for another’s selfish behavior affect observers’ decision-making by influencing their norm perceptions of the relevant behavior. We used modified dictator games, in which the dictator decides how to divide a small amount of money between himself or herself and another individual (the receiver). The receiver (the second party) or a third party may inflict a punishment on the dictator. In Experiment 1, participants were told that a dictator had unfairly distributed the money and that either the second or third party reacted by verbally blaming the dictator. In Experiment 2, participants were told that either the majority of second or third parties chose to inflict monetary punishment when facing an unfair dictator. The results consistently showed that compared with SPP, TPP induced a belief in observers that the selfish behavior was less common and acceptable. In addition, the altered descriptive norm perception (but not injunctive norm perception) further influenced individuals’ own sharing behaviors.

## Experiment 1

### Methods

#### Ethics

The current research was approved by the ethics committee of the Department of Psychology and Behavioral Sciences of Zhejiang University. All the participants in both Experiment 1 and Experiment 2 provided written informed consent in accordance with the Declaration of Helsinki.

#### Participants

One hundred and twenty-two undergraduate students at Zhejiang University (41 men, 81 women; *M*_age_ = 20.19 years, *SD* = 1.82) participated in the experiment for monetary compensation of CNY 5 (about USD 0.75). They were allowed to keep any money they gained by playing the dictator game (a maximum of CNY 10).

#### Procedure

Participants were randomly assigned to SPP (*n* = 61) and TPP (*n* = 61) groups. After being seated in individual cubicles, the participants read instructions provided in paper handouts. To manipulate SPP and TPP, participants were introduced to the modified dictator game, in which the dictator can choose to split a small amount of money with the receiver, and the receiver (SPP group) or a third party (TPP group) can send verbal feedback to the dictator. In addition, we asked participants to view messages ostensibly obtained from previous studies (explained more fully below), Afterwards, participants answered a set of questions unrelated to the current study, which took about 10 minutes to complete. Subsequently, they individually played a one-shot dictator game with no risk of punishment, from which data regarding their norm perceptions and sharing behaviors were collected.

#### SPP and TPP manipulations

In this game, the dictator (Player A) was given 100 points (10 points equals CNY 1) and was allowed to give 0, 10, 20, 30, 40, or 50 points to the receiver (Player B). In the SPP condition, Player B was not given any points, and, in the TPP condition, a third party (Player C) was given 50 points. Player B in the SPP group and the Player C in the TPP group could send an unrestricted message to Player A after learning of Player A’s transmission amount. All players’ identities and game moves were anonymous, and all participants were allowed to keep the money they gained during the game.

The participants subsequently viewed the messages from Player B or C to Player A after being told that the messages had been randomly chosen from a previous study. All of the participants were told that Player A had transferred 20 points to Player B and that the message, from either Player B or C, read: “You are selfish. You shouldn’t think only of yourself.” To cover the study objectives, the participants were asked to indicate the extent to which they agreed or disagreed, based on the message, that either Player B or C was angry, happy, or sad. This information was used as a filler question that was later omitted from analysis.

#### Norm perceptions and sharing behaviors

Participants were asked to perform an anonymous dictator task with another student. In the task, Player A could transfer 0, 10, 20, 30, 40, or 50 points to Player B without a threat of punishment. Based on a previous study [18], we measured norm perceptions as follows. The participants estimated the percentage of students would transfer 0, 10, 20, 30, 40, or 50 points in the role of Player A (descriptive norm) and the percentage of students who would think that Player A should transfer 0, 10, 20, 30, 40, or 50 points (injunctive norm). Average estimated percentages were calculated for each type of social norm.

We required all of the participants to choose the number of points they would share with Player B. Each participant was informed that she or he would be paired with another participant and randomly assigned to a role in the game (Player A or B). Because both players in the game remained anonymous, participants would feel that their choices as Player A would remain unknown.

### Results

#### Norm perceptions and sharing behaviors

**Fig 1:**
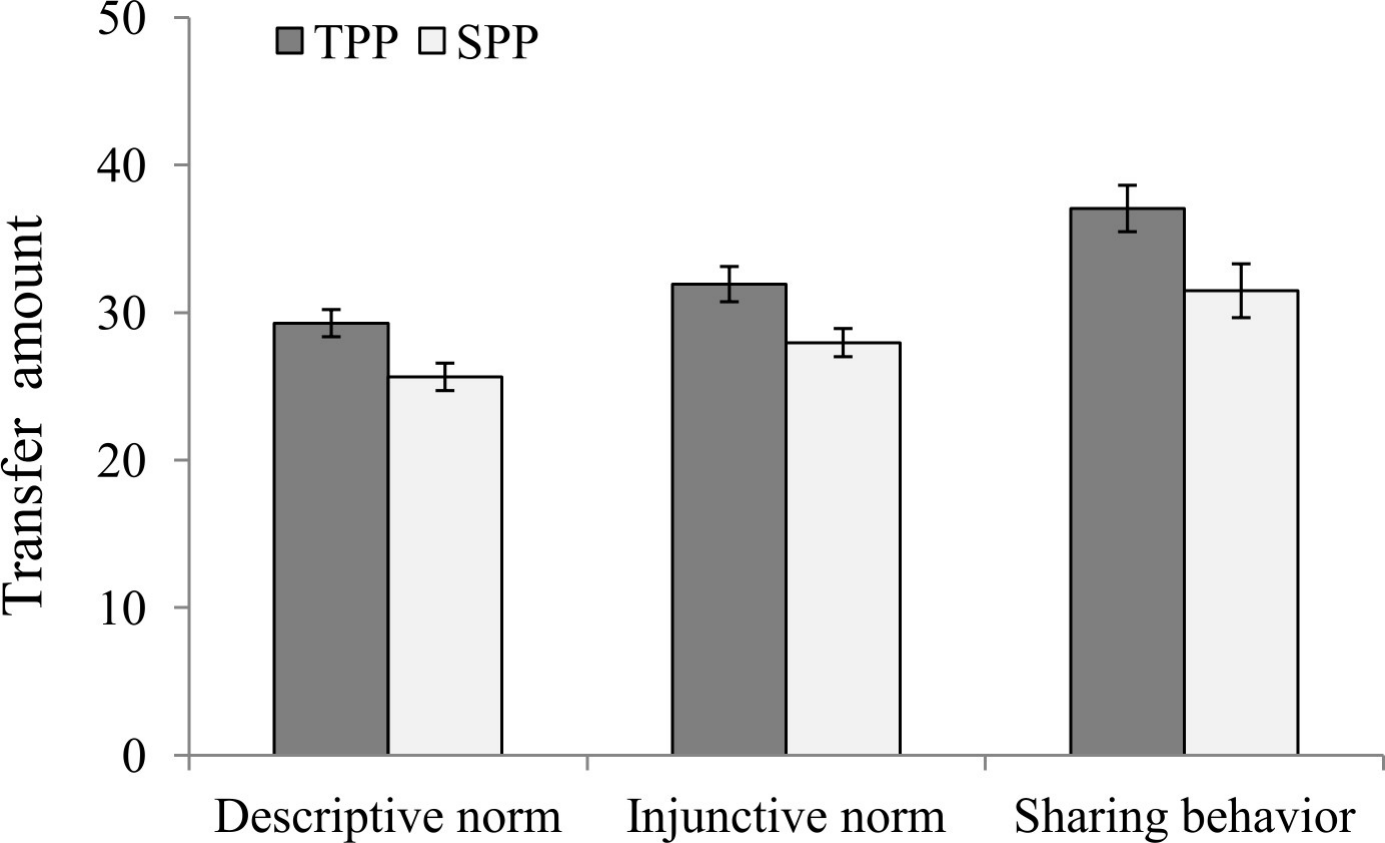
Descriptive and injunctive norm perceptions and sharing behaviors by group in Experiment 1 (error bars represent standard errors).

Independent group t-tests revealed significant differences between the SPP and TPP groups in descriptive norm perception, namely beliefs about the transfer amount of other students, (*t*(120) = 2.78, *p* = 0.006, Cohen’s *d* = 0.50, 95%*CI* = [0.14, 0.86]) and injunctive norm perception, namely beliefs about the transfer amount that other students would approve of (*t*(120) = 2.59, *p* = 0.011, Cohen’s *d* = 0.47, 95%*CI* = [0.11, 0.83]); see Fig 1. Participants in the TPP group reported a higher mean transfer amount for descriptive norms than those in the SPP group (*M*_*TPP*_ = 29.28, *SD* = 7.19, *M*_*SPP*_ = 25.64, *SD* = 7.26); furthermore, they estimated a higher mean transfer amount for injunctive norms than those in the SPP group (*M*_*TPP*_ = 31.92, *SD* = 9.32, *M*_*SPP*_ = 27.96, *SD* = 7.45). In addition, we found a significant group difference in sharing behavior (*t*(120) = 2.31, *p* = 0.022, Cohen’s *d* = 0.42, 95%*CI* = [0.06, 0.78]), in that the Player A participants in the TPP group shared more money than did those in the SPP group (*M*_*TPP*_ = 37.05, *SD* = 12.29, *M*_*SPP*_ = 31.48, *SD* = 14.24). For the multiple comparisons problem, we calculated the harmonic mean p-value that could be interpreted directly when the number of tests are small [19]. The results showed that overall the effect of the punishment source was significant (*p* = 0.010). We also conducted non-parametric tests (Mann–Whitney U test) to further confirm our findings. The results did not change.

#### Mediation analysis

**Fig 2:**
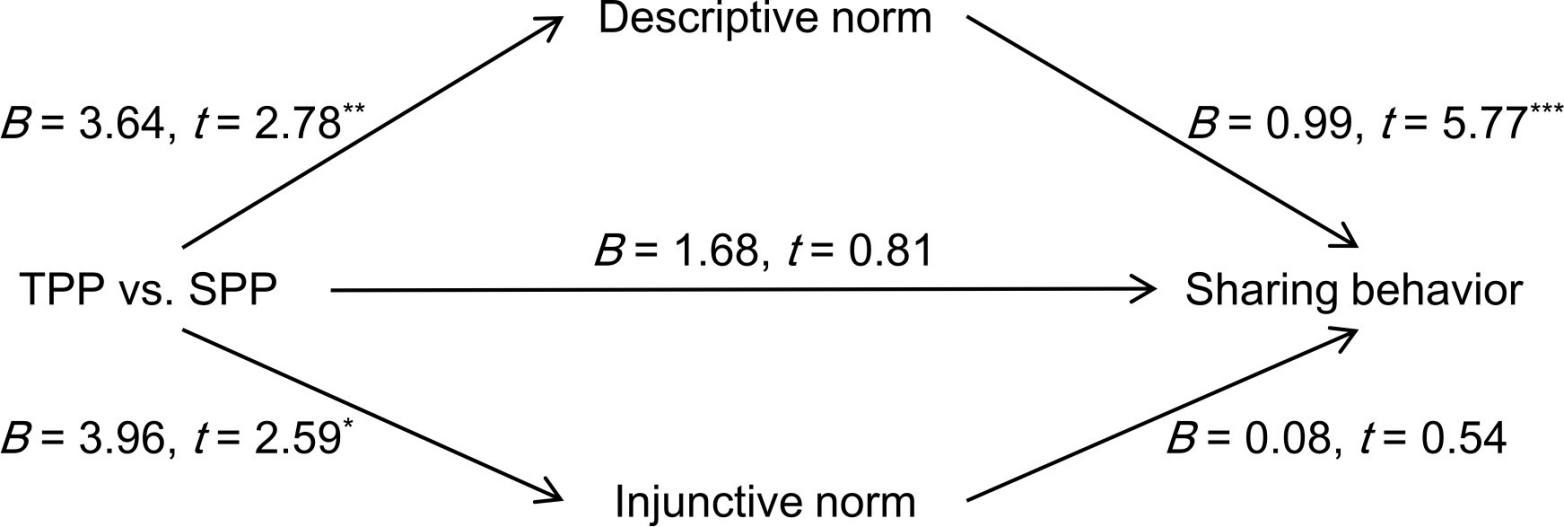
The influence of punishment source on observers’ sharing behaviors mediated by norm perceptions in Experiment 1 (* *p* < 0.05, ** *p* < 0.01, *** *p* < 0.001).

We used the bootstrapping procedure developed by Preacher and Hayes [20] to verify the hypothesized mediation effect of norm perceptions. The results are summarized in Fig 2. The TPP group reported a higher transfer amount than did the SPP group for descriptive norm perception (*B* = 3.64, *SE* = 1.31, 95%*CI =* [1.05, 6.23], *t* = 2.78, *p* = 0.006) and injunctive norm perception (*B* = 3.96, *SE* = 1.53, 95%*CI =* [0.94, 6.99], *t* = 2.59, *p* = 0.011). Participants’ sharing behaviors were significantly related to their perception of descriptive norms (*B* = 0.99, *SE* = 0.17, 95%*CI =* [0.65, 1.32], *t* = 5.77, *p* < 0.001); the indirect effect of descriptive norm perception did not include zero (*effect* = 3.59, *SE* = 1.36, 95% *bootstrap CI* = [1.33, 6.80]). However, participants’ perception of injunctive norms did not predict sharing behaviors (*B* = 0.08, *SE* = 0.15, 95%*CI =* [-0.21, 0.37], *t* = 0.54, *p* = 0.592); the indirect effect of injunctive norm perception included zero (*effect* = 0.31, *SE* = 0.81, 95% *bootstrap CI* = [-1.12, 2.23]). Adding gender as a covariate did not change the main results; however, female participants shared more money with their partner than did male participants (*B* = 4.64, *SE* = 2.11, 95%*CI =* [0.47, 8.82], *t* = 2.20, *p* = 0.029).

### Discussion

Experiment 1 confirmed that TPP of another’s unfair transfer of money had a more positive influence than did SPP on observers’ descriptive and injunctive norm perceptions. In addition, the results showed that the altered descriptive, but not injunctive, norm perceptions further influenced participants’ personal decisions. Player A participants in the TPP group who believed in social norms of a higher transfer level shared more money than did their counterparts in the SPP group with their Player B partners. In other words, TPP of another’s selfish behavior did a better job than SPP in restraining observers from committing a selfish act because TPP expressed more positive social norms.

## Experiment 2

### Methods

#### Participants

One hundred and twenty first-year students (67 men, 53 women; *M*_age_ = 18.21 years, *SD* = 0.45) participated in the experiment for extra course credit and a chance to win a lottery. Written informed consent was obtained from all of the participants after the experiment was fully explained to them.

#### Procedure

Participants arrived at the laboratory and attended a short online survey in groups of approximately 30. The general procedure was similar to that in Experiment 1. The participants were randomly assigned to SPP (*n* = 58) and TPP (*n* = 62) groups and manipulated regarding the SPP and TPP. Subsequently, the participants individually played a one-shot dictator game with no risk of punishment, from which data regarding their norm perceptions and sharing behaviors were collected.

#### SPP and TPP manipulations

The participants were told that we had conducted another study previously. In that study, two or three students played a game for one shot. In the SPP group, participants were further told that, in the game, the dictator (Player A) was given CNY 10 and allowed to distribute it with the recipient (Player B). The dictator could either distribute the money unfairly (Option 1: the dictator kept CNY 8 whereas the recipient received CNY 2), or distribute it fairly (Option 2: the dictator and recipient each received CNY 5). The recipient could choose to intervene, only if the dictator had chosen the unfair option, which would cost the recipient CNY 1 and deduct the dictator’s payoff by CNY 3. In the TPP group, there was only one difference: rather than the recipient, a third party (Player C) who was paid a fixed amount of money could choose to intervene. We informed participants that when the dictator chose the unfair distribution, the majority of the recipients or third parties, depending on the group, had chosen to intervene. To enhance our manipulation of SPP and TPP, participants were asked to estimate the percentage of the recipients or third parties who had chosen to intervene.

#### Norm perceptions and sharing behaviors

Participants were asked to perform an anonymous dictator task with another student. In the task, both students would receive five lottery tickets. The student who was randomly chosen as the dictator (Player A) could freely distribute an additional 10 lottery tickets with the recipient (Player B). Each lottery ticket represented an individual opportunity to win a monetary prize (a big or small prize: CNY100 or 20). We included one big prize and two small prizes for each experiment session.

Participants were asked to estimate the average amount of lottery tickets (0, 1, 2, 3, 4 or 5) that Player A would give to Player B (descriptive norm) and the average amount that participants thought Player A should have given to Player B (injunctive norm). We also asked participants to indicate the amount of lottery tickets (0, 1, 2, 3, 4 or 5) that they thought Player A should have given to Player B as a measure of personal norm. Finally, we informed each participant that she or he would be paired with another participant and randomly assigned to a role in the game (Player A or B). All participants were required to indicate the amount of lottery tickets they would share with Player B.

### Results

#### Norm perceptions and sharing behaviors

**Fig 3:**
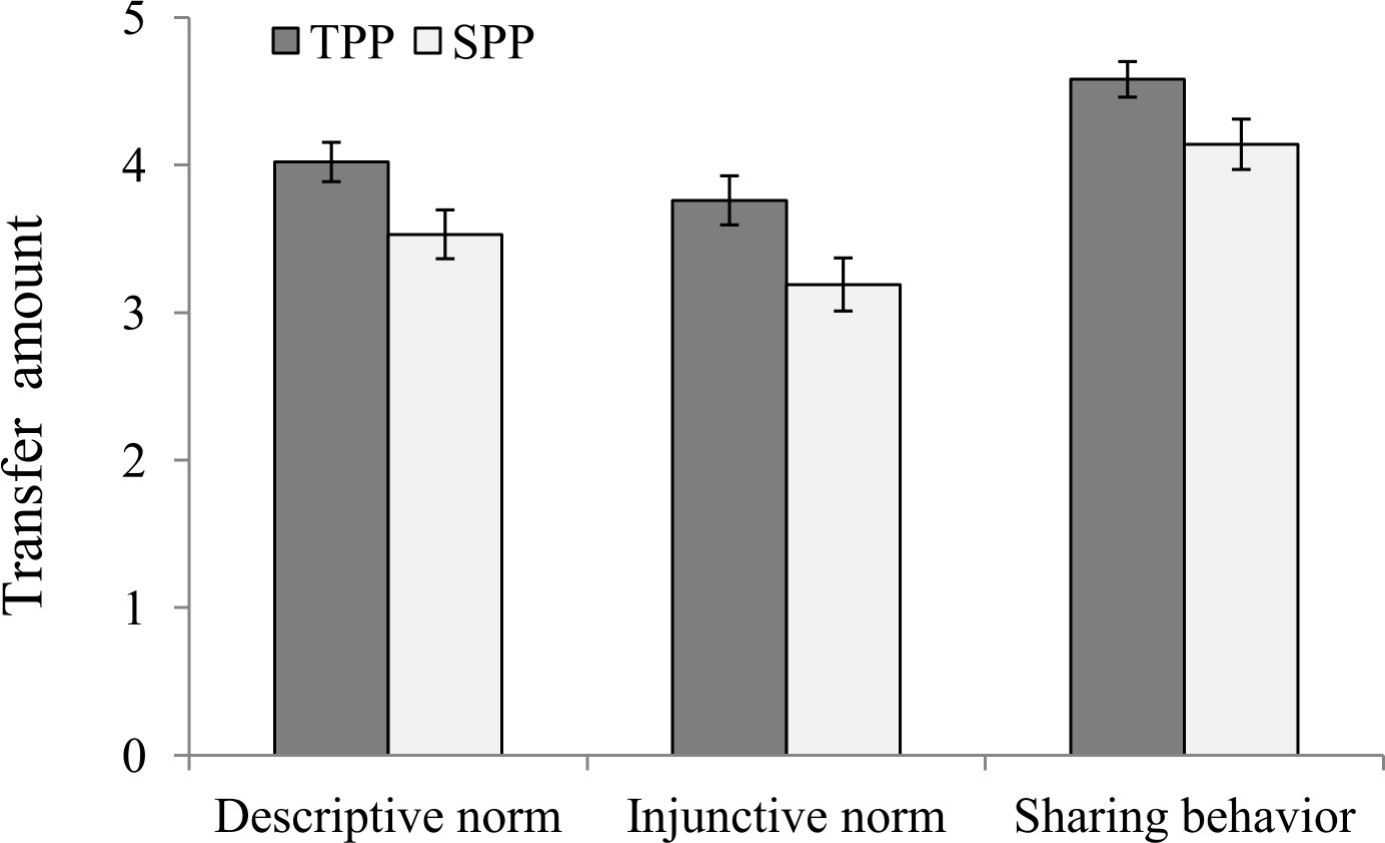
Descriptive and injunctive norm perceptions and sharing behaviors by group in Experiment 2 (error bars represent standard errors).

Consistent with the findings in Experiment 1, we found significant differences between the SPP and TPP groups in descriptive norm perception (*t*(118) = 2.28, *p* = 0.024, Cohen’s *d* = 0.42, *95%CI* = [0.06, 0.79]) and injunctive norm perception (*t*(118) = 2.32, *p* = 0.022, Cohen’s *d* = 0.43, *95%CI* = [0.06, 0.79]); see Fig 3. Participants in the TPP group reported a higher mean transfer amount for descriptive norms than did those in the SPP group (*M*_*TPP*_ = 4.02, *SD* = 1.05; *M*_*SPP*_ = 3.53, *SD* = 1.26); they also estimated a higher mean transfer amount for injunctive norms than did those in the SPP group (*M*_*TPP*_ = 3.76, *SD* = 1.31; *M*_*SPP*_ = 3.19, *SD* = 1.37). Further, there was a significant group difference in sharing behaviors (*t*(118) = 2.14, *p* = 0.035, Cohen’s *d* = 0.39, *95%CI* = [0.03, 0.75]) in that the Player A participants in the TPP group shared more lottery tickets with the Player B participants than did those in the SPP group (*M*_*TPP*_ = 4.58, *SD* = 0.95; *M*_*SPP*_ = 4.14, *SD* = 1.30). Again, we calculated the harmonic mean p-value, which remained significant (*p* = 0.026). Using non-parametric tests (Mann–Whitney U test) did not change the results.

#### Mediation analysis

**Fig 4:**
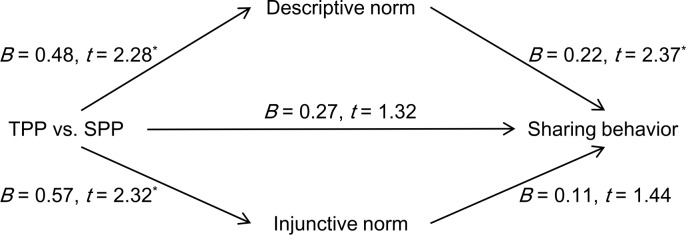
The influence of punishment source on observers’ sharing behaviors mediated by norm perceptions in Experiment 2 (* *p* < 0.05).

We used the bootstrap method to test the mediation effect of norm perceptions in the relationship between punishment source and observers’ sharing behaviors; see Fig 4. We found that the TPP group reported a higher sharing level than did the SPP group for descriptive norm perceptions (*B* = 0.48, *SE* = 0.21, *95%CI* = [0.06, 0.90], *t* = 2.28, *p* = 0.024) and injunctive norm perceptions (*B* = 0.57, *SE* = 0.25, *95%CI* = [0.08, 1.05], *t* = 2.32, *p* = 0.022). The participants’ sharing behaviors were significantly related to their descriptive norm perceptions (*B* = 0.22, *SE* = 0.09, *95%CI* = [0.04, 0.40], *t* = 2.37, *p* = 0.020). The indirect effect of descriptive norm perceptions did not include zero (*effect* = 0.11, *SE* = 0.07, *95% bootstrap CI* = [0.01, 0.30]). However, we did not find a significant effect of injunctive norm perception on sharing behaviors (*B* = 0.11, *SE* = 0.08, *95%CI* = [-0.04, 0.27], *t* = 1.44, *p* = 0.153). The indirect effect of injunctive norm perception was smaller (*effect* = 0.06, *SE* = 0.05, *95% bootstrap CI* = [0.00, 0.23]). Adding gender and personal norm as covariates did not change the main results, although participants’ personal norm also positively predicted sharing behaviors (*B* = 0.19, *SE* = 0.06, *95%CI* = [0.06, 0.32], *t* = 2.95, *p* = 0.004).

### Discussion

In Experiment 2, using a different method to manipulate SPP and TPP, we showed that compared with learning that a majority of second parties had punished another’s unfair transfer, learning that a majority of third parties had punished the same unfair transfer would make observers believe that a higher transfer level was both the descriptive and injunctive norm. The results also showed that people’s sharing behaviors were related to their perceptions of the descriptive, but not injunctive norm, which was consistent with the findings in Experiment 1. Notably, the average sharing level was relatively high in Experiment 2. One potential reason may have been that the incentive was small. It has been shown that people are more generous when the incentive is small than when it is large [21]. Nevertheless, the findings in Experiment 2 further confirmed that TPP of another’s selfish behavior communicates more positive normative information and better restrains observers from engaging in selfish behaviors than does SPP.

## General discussion

Communicating social norms is proposed to be a crucial function of punishment. This study contributes empirical evidence that the effectiveness of punishment for expressing positive social norms depends on the source of punishment. Through two experiments, we consistently found that observers had more positive descriptive norm perceptions (beliefs about the transfer amount of others) and injunctive norm perceptions (beliefs about the transfer amount that others would approve of) when punishment was inflicted by a third party than when it was inflicted by an injured second party. Further, we found that the source of punishment shaped the observers’ norm perception (especially descriptive norm perception), which, in turn, influenced their personal decisions. Those who were told of TPP regarding an unfair transfer of money shared more money or lottery tickets with others in the game than did those who were told of SPP regarding unfair sharing.

Our results are in accordance with previous studies showing that self-serving motives would undermine the norm expressive function of punishment [22]. When third parties profit from punishing selfish behaviors, TPP ceases to signal positive social norms and might even increase selfish behaviors [22]. The motives for punishment might be questioned even when the punishers do not seem to directly profit from punishing the transgression, such as the case for SPP [14]. The current data confirmed that TPP communicated more positive social norms for the relevant behaviors than did SPP, which was believed to be driven by mixed motives.

Whereas it is widely agreed that norm perceptions have a powerful influence on individuals’ decision-making [9], the relative importance of descriptive norm and injunctive norms is still debated [18, 23]. In addition, some researchers have suggested that the sharing behaviors in the dictator game are largely determined by a personal preference for morality [24, 25]. Our results showed that both descriptive norms and individuals’ personal norms, but not injunctive norms, predicted their sharing behaviors. One crucial reason for compliance with injunctive norms is to gain social approval [26]. Future research should consider investigating whether injunctive norms have a stronger impact on individuals’ behaviors in specific situations, such as making decisions publicly.

Our findings suggest that TPP and SPP might promote prosocial behaviors in different ways. Punishments might restrain individuals from committing selfish behaviors both by reducing the perceived rewards of the undesired behaviors [1] and by influencing their perceptions of the relevant social norms [9]. When confronted with selfish behaviors, second parties tend to punish them more often and more severely than to third parties [10, 27]. Consequently, SPP might be a stronger deterrent compared to TPP; however, we found that TPP had a stronger influence than did SPP as a source of information about positive social norms. Because influencing individuals’ norm perceptions effectively changes their personal behaviors, TPP, rather than SPP, is likely to inhibit selfish behaviors by its relatively strong influence on the perception of relevant social norms and their associated behaviors.

One limitation of the current study is that the sample sizes are relatively small (approximately 120 for each experiment), which is close to the minimum sample size required to detect a medium level of mediated effect [28]. Nevertheless, the consistent findings of the two experiments provide preliminary evidence supporting our hypothesis. Future studies should consider replicating our findings with a larger sample size.

## Conclusion

In sum, the source of punishment is an important influence on the norm expressive function of punishment. Compared with SPP, TPP communicates more positive information regarding the descriptive and injunctive norms of the relevant behavior. Observers’ decision-making is affected by their norm perception, especially the perception of descriptive norms. Consequently, compared to SPP, TPP might better inhibit observers’ selfish behaviors by shaping norm perceptions, particularly when there is no threat of punishment.

## Supporting information

S1 File Data. https://osf.io/bnvxj/?view_only=27a61915dec34aa49977d2ba898ee6b1
(ZIP)
